# Synthesis, spectral characterization, and biological studies of 3,5-disubstituted-1,3,4-oxadiazole-2(3H)-thione derivatives

**DOI:** 10.3906/kim-2008-44

**Published:** 2021-06-30

**Authors:** Tuğçe ÖZYAZICI, Fikrettin ŞAHİN, Meriç KÖKSAL

**Affiliations:** 1 Department of Pharmaceutical Chemistry, Faculty of Pharmacy, Yeditepe University, İstanbul Turkey; 2 Department of Pharmaceutical Chemistry, Faculty of Pharmacy, Sağlık Bilimleri University, İstanbul Turkey; 3 Department of Genetics and Bioengineering, Faculty of Engineering, Yeditepe University, İstanbul Turkey

**Keywords:** 1,3,4-Oxadiazole, piperidine, antibacterial activity, cytotoxicity

## Abstract

The reaction of 3,4-dichlorophenyl-1,3,4-oxadiazole-2( *3H* )-thione with piperidine derivatives via Mannich reaction was used to generate eleven novel compounds in moderate to good yields. Synthesized molecules were characterized according to their structure with ^1^H NMR, ^13^C NMR and FT-IR spectral foundations, which were compatible with literature informations. Antimicrobial activity and cytotoxicity studies were done by disc diffusion and NCI-60 sulphordamine B assay methods. The antimicrobial test results revealed that synthesized compounds have better activity against gram-positive species than gram-negative ones. A total analysis of the antibacterial, antifungal, and antiyeast activity revealed that newly synthesized compounds were really active against *Bacillus cereus* , *Bacillus ehimensis,* and *Bacillus thuringiensis* species *.* For cytotoxicity, among three different cancer cell lines (HCT116, MCF7, HUH7) compounds **5c, 5d, 5e, 5f, 5g, 5i, 5j** and **5k** were seemed especially effective on HUH7 cancer cell line via moderate to good activity. More significantly, against liver carcinoma cell line (HUH7) most of the compounds of the series ( **5c-5g** and **5i-5j** ) have better IC_50_ values (IC_50_= 18.78 µM) than 5-Florouracil (5-FU) and also compound **5d** possessed 10.1 µM value, which represents good druggable cytotoxic activity. Further, the molecules were also screened for in silico chemoinformatic and toxicity data to gather the predicted bioavailibity and safety measurements.

## 1. Introduction 

Antimicrobial drugs are known as a group of pharmaceuticals that preserve various defensive effects against bacteria, fungi, virus, and parasites. It is well recognized that many antimicrobial agents are necessary to treat life-threatening infections, but improved bacterial resistance against these medications may cause worser consequences for human health. Antimicrobial resistance occurs naturally over time, when responsible strains of a microorganism exchange among microorganisms usually through genetic material. This proceeding problem of currently marketed antimicrobials have speed up the birth of new variants of mechanisms about resistance and fairly fast rise in the number of microorganisms which spread among all over the world [1–4]. 

To prevent the risk of increase in antimicrobial resistance, different therapy strategies should be applied according to the type of microorganisms (drug-resistant or not) and disease severity. Since 2005, World Health Organization (WHO) has developed a criteria to order antimicrobials according to their relative importance in human medicine and the study has been used by clinicians to preserve the usage quality of currently available drugs [5]. This commercially available medicines which are used for infection and cancer treatments have a common point: the emergence of resistance against multiple drugs [6,7]. Other related problems are insufficient selectivity and undesirable side effects consequences of the patients [6,8]. Hence, there is a massive requirement for the improvement of new antimicrobial and anticancer therapies, with higher selectivity, besides serving fewer side effects than current ones. So, the main goal is resistance prevention, good potential of action, and last but not least, diminished side effects [9–12]. As well as developing antimicrobial treatment strategies, new studies on antimicrobials have been maintained as cancer theraphy due to strong evidences about the relationship between microbial diseases and cancer. This data supports the combined usage of antimicrobial and anticancer medications in clinical area. In addition, studies on direct antiproliferative activity of certain antimicrobials and prophylactic effect of antimicrobials against post-chemotheraphy infections due to immunosupression are being discussed with their possible mechanisms about cancer treatment [13]. Today, it is reported in the literature that antiproliferative activity of clinically used antimicrobials is related to the topoisomerase enzyme inhibition [14,15], degradation of tumorigenic proteins [16,17], destabilization [18], antiangiogenic effects and apoptosis [19–24].

1,3,4-Oxadiazole is generally used entity for pharmacophore development and has been investigated because of its good metabolic profile and hydrogen-bonding capacity within the receptor site. Presence of azole group (N=C-O) also elevates lipophilicity feature of compound, which provides advantage for its transportation through cell membrane to reach the target site and show various biological activities [25]. These co-operative properties provide great benefits to obtain desired antimicrobial and anticancer activity within various proven in vitro and in vivo models. In 2013, Du and coworkers dealed with modeling 1,3,4-oxadiazole ring to obtain both of anticancer and antimicrobial effect by targeting thymidylate synthase (TS), which is an important enzyme for DNA synthesis. Newly synthesized compounds were identified as potent inhibitors against two kinds TS proteins with IC_50_ values of 0.47–1.4 µM [26].

Nowadays, dual antimicrobial-anticancer activity of 1,3,4-oxadiazole core structure has been an important concern. Ahsan et al. dealed with disubstituted derivatives of 1,3,4-oxadiazole with antimicrobial-anticancer activity capacity ,and synthesized analogs showed moderate to severe potency for this binary physiological topics [27]. In another research, Savariz and coworkers studied with 3,5-disubstituted-1,3,4-oxadiazole pharmacophore group, which were derived with different functional moieties via Mannich reaction. Results showed that both anticancer and antimicrobial activity of synthesized series have intermediate to excellent effect, and especially one of the compounds, which has a heterocyclic ring from third position of core structure, improved antitumor activity, which was found to be 4.5 timefold compared to the precursor molecule [28]. Under the shadow of previous experiences, Selvaraj and coworkers synthesized fifteen 1,3,4-oxadiazole derivatives, and they were found to have moderate to severe inhibitor effect against different microbial and cancer cell lines in 2017 [29].

Based on the statements above, to develop new, potent antimicrobial and anticancer agents, we aimed to synthesize a new series of 3,5-disubstituted-1,3,4-oxadiazole-2( *3H* )-thione derivatives carrying different piperidine side chains. The compounds were evaluated for their antimicrobial and cytotoxicity profile to investigate the effect of molecular variations on activity against different bacteria, fungi, and yeasts. Further, compounds were tested in against various cancer cell lines for their cytotoxic activity. 

## 2. Experimental

### 2.1. Materials and measurements

Melting points of compounds were checked by Mettler Toledo FP62 capillary melting point apparatus (Mettler-Toledo, Greifensee, Switzerland) and are uncorrected. Infrared spectral data were obtained by the use of Perkin-Elmer Spectrum One series FT-IR apparatus (Version 5.0.1) (Perkin Elmer, Norwalk, CT, USA), with potassium bromide pellets and the frequencies were presented in cm^–1^. The ^1^H-NMR spectra were checked via Varian Mercury-400 FT-NMR spectrometer (Varian, Palo Alto, CA, USA) using tetramethylsilane as the internal reference, with dimethyl Sulfoxide (DMSO-d_6_), as solvent, the chemical shifts were reported in parts per million (ppm), and coupling constants (J) were given in hertz (Hz). Elemental analyses were done by LECO 932 CHNS instrument (Leco-932, St. Joseph, MI, USA) and were within ± 0.4% of the theoretical values.

### 2.2. Chemistry

#### 2.2.1. General procedure for the synthesis of 5-(3,4-dichlorophenyl)-1,3,4-oxadiazole-2(3H)-thione (4)

Solution of aroyl hydrazine (3.13 mmol) and carbon disulfide (6.27 mmol) in absolute ethanol (15 mL) were mixed in cold media (0 °C) and after the addition of potassium hydroxide (3.13 mmol) in one portion, the mixture was refluxed for 8 h. After the reaction was over, solvent was evaporated and the residue was acidified with 2M hydrochloric acid and extracted with ethyl acetate (2 × 20 mL). The organic layers were washed with water and dried with anhydrous sodium sulphate. Filtration and concentration in vacuo gave a solid, which was recrystallized from ethanol to give the compound [30].

#### 2.2.2. General procedure for the synthesis of 5-(3,4-dichlorophenyl)-3-[(substitutedpiperidine)methyl]-1,3,4-oxadiazole-2(3H)-thione derivatives (5a-5k) 

A mixture of 5-(3,4-dichlorophenyl)-1,3,4-oxadiazole-2(3 *H* )-thione (0.71 g, 3 mmol), an appropriate N-substituted amine (3 mmol) and 37% formaldehyde solution (1 mL) in ethanol (15 mL), was refluxed 3–5 h. The crude products were either precipitated or it was necessary to add water in case of not precipitated. The crude products were filtered, washed with water, dried, and crystallized from ethanol or ethanol/water. 

##### 2.2.2.1. 5-(3,4-Dichlorophenyl)-3-[(4-phenylpiperidin-1-yl)methyl]-1,3,4-oxadiazole-2(3H)-thione (5a)

White powder, yield 87%, Mp 182.0°C; FT-IR (KBr) ν_max_ : 3075-3024 (Aromatic C-H), 1611 (C=N), 1435 (C=C), 1318 (C=S), 1238 (C-O-C) cm^–1^; ^1^H-NMR (DMSO- *d* *_6_* , 400 MHz) ppm: δ = 8.07 (1H, s, phenyl H_2_), 7.79 (1H, d, *J* =10 Hz, phenyl H_5_), 7.61 (1H, bd, *J* = 8.8 Hz, phenyl H_6_), 7.34-7.19 (5H, m, phenyl H_2_’+H_3_’+H_4_’+H_5_’+H_6_’), 5.11 (2H, s, -C **H**_2_-), 3.16 (2H, bd, *J* =11.6 Hz, piperidine H_2_), 2.65 (2H, t, *J* =10.8 Hz, piperidine H_6_), 1.76 (2H, bd, *J* =11.2 Hz, piperidine H_3_), 1.65 (1H, m, piperidine H_4_), 1.66–1.62 (2H, m, piperidine H_5_); C^13^-NMR (100 MHz, DMSO) *δ* 179.9 ( **C** =S), 159.8 ( **C** =N), 151.5 ( **C** **_1_** **^’^** , phenyl), 134.9 ( **C** **_4_** , phenyl), 132.4 ( **C** **_3_** , phenyl), 131.8 ( **C** **_5_** , phenyl), 128.7 ( **C** **_3_** **’+C** **_5_** **’** , phenyl), 126.7 ( **C** **_4_** **^’^** , phenyl), 126.2 ( **C** **_2_** , phenyl), 126.1 ( **C** **_6_** , phenyl), 125.1 ( **C** **_2_** **’+C** **_6_** **’** , phenyl), 122.1 ( **C** **_1_** , phenyl), 73.5 (N- **C** H_2_-N), 69.0 ( **C** **_3_** , piperidine), 46.2 ( **C** **_1_** **+C** **_5_** , piperidine), 37.7 ( **C** **_2_** **+C** **_4_** , piperidine); Anal. Calcd. for C_20_H_19_Cl_2_N_3_OS: C, 57.15; H, 4.56; N, 10.00; S, 7.63. Found: C, 57.32; H, 4.56; N, 10.14; S, 7.66.

##### 2.2.2.2. 5-(3,4-Dichlorophenyl)-3-[(4-hydroxy-4-phenylpiperidin-1-yl)methyl]-1,3,4-oxadiazole-2(3H)-thione (5b)

White powder, yield 69.23%, Mp 182.1°C; FT-IR (KBr) ν_max_ : 3456 (O-H), 3077 (Aromatic C-H), 1435 (C=N), 1417 (C=C), 1317 (C=S), 1235 (C-O-C) cm^–1^; ^1^H-NMR (DMSO- *d* *_6_* , 400 MHz) ppm: δ = 8.06 (1H, s, phenyl H_2_), 7.86 (2H, d, *J* = 1.2 Hz, phenyl H_5_+H_6_), 7.46 (2H, d, *J* = 8 Hz, phenyl H_2_^’^+H_6_^’^), 7.36–7.18 (3H, m, phenyl H_3_^’^+H_4_^’^+H_5_^’^), 5.10 (2H, s, N-C **H**_2_-N), 4.79 (1H, bs, O **H** ), 2.94 (4H, t, *J* = 9.6 Hz, piperidine H_2_+H_6_), 1.93-1.91 (2H, m, piperidine H_5_), 1.59 (2H, d, *J* = 12 Hz, piperidine H_3_,); C^13^-NMR (100 MHz, DMSO) *δ* 177.7 ( **C** =S), 156.6 ( **C** =N), 149.7 (phenyl **C** **_1_** **^’^** ), 134.9 (phenyl **C** **_4_** ), 132.4 (phenyl **C** **_3_** ), 131.8 (phenyl **C** **_5_** ), 127.7 (phenyl **C** **_3_** **’+C** **_5_** **’** ), 127.6 (phenyl **C** **_4_** **^’^** ), 126.1 (phenyl **C** **_6_** ), 126.1 (phenyl **C** **_2_** ), 124.6 (phenyl **C** **_2_** **’+C** **_6_** **’** ), 122.8 (phenyl **C** **_1_** ), 73.5 (piperidine **C** **_3_** ), 69.0 (N- **C** H_2_-N), 46.2 (piperidine **C** **_1_** **+C** **_5_** ), 37.7 (piperidine **C** **_2_** **+C** **_4_** ); Anal. Calcd. for C_20_H_19_Cl_2_N_3_O_2_S: C, 55.05; H, 4.39; N, 9.63; S, 7.35. Found: C, 54.85; H, 4.27; N, 9.72; S, 7.42.

##### 2.2.2.3. 5-(3,4-Dichlorophenyl)-3-[(4-acetyl-4-phenylpiperidin-1-yl)methyl]-1,3,4-oxadiazole-2(3H)-thione (5c)

White powder, yield 52.48%, Mp 149.8°C; FT-IR (KBr) ν_max_ : 2924-2831 (Aliphatic C-H), 1698 (C=O), 1606 (C=N), 1422 (C=C), 1330 (C=S), 1244 (C-O-C) cm^–1^; ^1^H-NMR (DMSO- *d* *_6_* , 400 MHz) ppm: δ = 8.01 (1H, d, *J* = 1.6 Hz, phenyl H_2_)_, _7.86–7.82 (2H, m, phenyl H_5_+H_6_), 7.38–7.31 (5H, m, aromatic H_2_^’^+H_3_^’^+H_4_^’^+H_5_^’^+H_6_^’^), 5.01 (2H, s, N-C **H**_2_-N), 2.92 (2H, d, *J* = 12.4 Hz, piperidine H_2_), 2.63 (2H, t, *J* =10.8 Hz, piperidine H_6_), 2.43 (2H, d, *J* = 11.2 Hz, piperidine H_5_), 1.97–1.89 (2H, m, piperidine H_3_), 1.84 (3H, s, CO-C **H**_3_); C^13^-NMR (100 MHz, DMSO) *δ* 208.6 ( **C** =O), 177.7 ( **C** =S), 156.8 ( **C** =N), 141.2 (phenyl **C** **_1_** **^’^** ), 134.8 (phenyl **C** **_4_** ), 132.4 (phenyl **C** **_3_** ), 131.8 (phenyl **C** **_5_** ), 128.7 (phenyl **C** **_3_** **^’^** **+C** **_5_** **^’^** ), 127.6 (phenyl **C** **_4_** **^’^** ), 127.0 (phenyl **C** **_6_** ), 126.2 (phenyl **C** **_2_** **+C** **_6_** ), 126.1 (phenyl **C** **_2_** ), 122.9 (phenyl **C** **_1_** ), 70.5 (N- **C** H_2_-N), 53.5 (piperidine **C** **_3_** ), 47.4 (piperidine **C** **_1_** **+C** **_5_** ), 32.1 (piperidine **C** **_2_** **+C** **_4_** ), 25.4 (CO **C** H_3_); Anal. Calcd. for C_22_H_21_Cl_2_N_3_O_2_S: C, 57.15; H, 4.58; N, 9.09; S, 6.93. Found: C, 57.35; H, 4.40; N, 9.03; S, 6.55.

##### 2.2.2.4. 5-(3,4-Dichlorophenyl)-3-[(4-cyano-4-phenylpiperidin-1-yl)methyl]-1,3,4-oxadiazole-2(3H)-thione (5d)

White crystals Yield 47.68%, , Mp 168.7°C; FT-IR (KBr) ν_max_ : 3091 (Aromatic C-H), 2931 (Aliphatic C-H), 2238 (CºN), 1607 (C=N), 1432–1415 (C=C), 1322 (C=S), 1249 (C-O-C) cm^–1^; ^1^H NMR (DMSO- *d* *_6_* , 400 MHz) ppm: δ = 8.10 (1H, s, phenyl H_2_), 7.89 (2H, d, *J* = 0.8 Hz, phenyl H_5_+H_6_), 7.53 (2H, d, *J* = 8.0 Hz, phenyl H_2_^’^+H_6_^’^), 7.44 (2H, t, *J* = 7.6 Hz, phenyl H_3_^’^+H_5_^’^), 7.37 (1H, t, *J* = 7.6 Hz, phenyl H_4_^’^), 5.13 (2H, s, N-C **H**_2_-N), 3.25 (2H, d, *J* = 12.4 Hz, piperidine H_2_), 2.87 (2H, t, *J* = 10.8 Hz, piperidine H_5_), 2.16 (2H, d, *J* = 12.4 Hz, piperidine H_6_), 2.03 (2H, t, *J* = 12.8 Hz, piperidine H_3_); C^13^-NMR (100 MHz, DMSO) *δ* 177.5 ( **C** =S), 156.8 ( **C** =N), 139.9 (phenyl **C** **_1_** ’), 135.1 (phenyl **C** **_4_** ), 132.4 (phenyl **C** **_3_** ), 131.8 (phenyl **C** **_5_** ), 129.0 (phenyl **C** **_3_** **^’^** **+C** **_5_** **^’^** ), 128.0 (phenyl **C** **_4_** **^’^** ), 127.7 (phenyl **C** **_6_** ), 126.2 (phenyl **C** **_2_** ), 125.5 (phenyl **C** **_2_** **^’^** **+C** **_6_** **^’^** ), 122.6 (phenyl **C** **_1_** ), 121.6 ( **C** ≡N), 70.1 (N- **C** H_2_-N), 47.5 (piperidine **C** **_1_** **+C** **_5_** ), 41.3 (piperidine **C** **_3_** ), 35.3 (piperidine **C** **_2_** + **C** **_4_** ); Anal. Calcd. for C_21_H_18_Cl_2_N_4_OS: C, 56.63; H, 4.07; N, 12.58; S, 7.20. Found: C, 56.54; H, 4.20; N, 12.57; S, 7.24.

##### 2.2.2.5. 3-[(4-Benzylpiperidin-1-yl)methyl]-5-(3,4-dichlorophenyl)-1,3,4-oxadiazole-2(3H)-thione (5e)

 White crystals, Yield 56.92%, Mp 94.8°C; FT-IR (KBr) ν_max_ : 3024 (Aromatic C-H), 2914 (Aliphatic C-H), 1617 (C=N), 1436-1418 (C=C), 1318 (C=S), 1233 (C-O-C) cm^–1^; ^1^H-NMR (DMSO- *d* *_6_* , 400 MHz) ppm: δ =8.04 (1H, s, phenyl H_2_), 7.84 (2H, d, *J* = 1.6 Hz, phenyl H_5_+H_6_), 7.25 (2H, t, *J* = 7.2 Hz, phenyl H_2_^’^+H_6_^’^), 7.14–7.11 (3H, m, phenyl H_3_^’^, H_4_^’^, H_5_^’^), 5.03 (2H, s, N-C **H**_2_-N), 3.01 (2H, d, *J* = 11.6 Hz, piperidine H_2_), 2.45 (2H, d, *J* = 6.8 Hz, N-C **H**_2_-Phenyl), 2.43 (2H, d, *J* = 11.6 Hz, piperidine H_6_), 1.54 (2H, d, *J* = 12 Hz, piperidine H_3_), 1.44–1.40 (1H, m, piperidine H_4_), 1.17 (2H, q, *J* = 10.6 Hz, piperidine H_5_); C^13^-NMR (100 MHz, DMSO) *δ* 177.8 ( **C** =S), 156.8 ( **C** =N), 140.1 (phenyl **C** **_1_** ’), 134.5 (phenyl **C** **_4_** ), 132.4 (phenyl **C** **_3_** ), 131.7 (phenyl **C** **_5_** ), 128.8 (phenyl **C** **_3_** ’ **+C** **_5_** ’), 128.0 (phenyl **C** **_2_** ’ **+C** **_6_** ’), 127.5 (phenyl **C** **_2_** ), 126.0 (phenyl **C** **_4_** ’), 125.7 (phenyl **C** **_6_** ), 123.2 (phenyl **C** **_1_** ), 71.0 (N- **C** H_2_-N), 50.0 (piperidine **C** **_1_** **+C** **_5_** ), 42.1 (N- **C** H_2_-Phenyl), 36.5 (piperidine **C** **_3_** ), 31.5 (piperidine **C** **_2_** **+C** **_4_** ); Anal. Calcd. for C_21_H_21_Cl_2_N_3_OS: C, 58.07; H, 4.87; N, 9.67; S, 7.38. Found: C, 58.24; H, 4.91; N, 9.85; S, 7.55.

##### 2.2.2.6. 5-(3,4-Dichlorophenyl)-3-{[4-(morpholin-4-yl)piperidin-1-yl]methyl}-1,3,4-oxadiazole-2(3H)-thione (5f)

White powder, yield 59.55%, Mp 156.8°C; FT-IR (KBr) ν_max_ : 2938 (Aromatic C-H), 1610 (C=N), 1448 (C=C), 1326 (C=S), 1246 (C-O-C) cm^–1^; ^1^H-NMR (DMSO- *d* *_6_* , 400 MHz) ppm: δ = 8.04 (1H, s, phenyl H_2_), 7.84 (2H, d, *J* = 2 Hz, phenyl H_5_+H_6_), 5.02 (2H, s, N-C **H**_2_-N), 3.58 (5H, bs, morpholine C **H**_2_O, piperidine H_4_), 3.33 (5H, bs, morpholine NC **H**_2_, piperidine H_2_), 3.09 (2H, d, *J* = 11.2 Hz, piperidine H_6_), 1.79 (2H, d, *J* = 11.2 Hz, piperidine H_3_), 1.42 (2H, bs, piperidine H_5_); C^13^-NMR (100 MHz, DMSO) *δ* 178.3 ( **C** =S), 157.2 ( **C** =N), 132.1 (phenyl **C** **_4_** ), 131.6 (phenyl **C** **_3_** ), 127.2 (phenyl **C** **_5_** ), 125.8 (phenyl **C** **_6_** ), 123.5 (phenyl **C** **_2_** ), 122.0 (phenyl **C** **_1_** ), 70.4 (N- **C** H_2_-N), 65.7 (morpholine **C** **_2_** **+C** **_3_** ), 63.0 (piperidine **C** **_3_** ), 60.8 (morpholine **C** **_1_** **+C** **_4_** ), 49.0 (piperidine **C** **_1_** **+C** **_5_** ), 27.1 (piperidine **C** **_2_** **+C** **_4_** ); Anal. Calcd. for C_18_H_22_Cl_2_N_4_O_2_S: C, 50.35: H, 5.16: N, 13.05; S, 7.47. Found: C, 49.61; H, 4.81; N, 12.92; S, 7.75.

##### 2.2.2.7. 1-{[5-(3,4-Dichlorophenyl)-2-thioxo-1,3,4-oxadiazol-3(2H)-yl]methyl}piperidine-4-carboxylic acid (5g)

White powder, yield 49.55%, Mp 181.3°C; FT-IR (KBr) ν_max_ : 3424 (O-H), 3042 (Aromatic C-H) 2941 (Aliphatic C-H), 1725 (C=O), 1554 (C=N), 1441–1419 (C=C), 1320 (C=S), 1239 (C-O-C) cm^–1^; ^1^H-NMR (DMSO- *d* *_6_* , 400 MHz) ppm: δ = 8.01 (1H, s, phenyl H_2_), 7.82 (2H, s, phenyl H_5_+H_6_), 5.04 (2H, s, N-C **H**_2_ **-** N), 3.00 (4H, t, *J* = 11.4 Hz, piperidine H_2_+H_6_), 2.00 (1H, d, *J* = 12.0 Hz, piperidine H_4_,), 1.82 (2H, d, *J* = 12.0 Hz, piperidine H_3_), 1.60–1.51 (2H, m, piperidine H_5_); C^13^-NMR (100 MHz, DMSO) *δ* 179.1 ( **C** =O), 175.7 ( **C** =S), 157.7 ( **C** =N), 133.4 (phenyl **C** **_4_** ), 132.1 (phenyl **C** **_3_** ), 131.6 (phenyl **C** **_2_** **+C** **_6_** ), 126.9 (phenyl **C** **_5_** ), 125.5 (phenyl **C** **_1_** ), 70.6 (N- **C** H_2_-N), 49.9 (piperidine **C** **_1_** **+C** **_5_** ), 42.5 (piperidine **C** **_3_** ), 27.6 (piperidine **C** **_2_** + **C** **_4_** ); Anal. Calcd. for C_15_H_15_Cl_2_N_3_O_3_S: C, 46.40; H, 3.89; N, 10.82; S, 8.26. Found: C, 47.42; H, 4.49; N, 10.88; S, 7.75.

##### 2.2.2.8. 1-{[5-(3,4-Dichlorophenyl)-2-thioxo-1,3,4-oxadiazol-3(2H)-yl]methyl}piperi-dine-3-carboxylic acid (5h)

White powder, yield 70.83%, Mp 181.7°C; FT-IR (KBr) ν_max_ : 3421 (O-H), 2934 (Aromatic C-H), 1710 (C=O), 1609 (C=N), 1437–1414 (C=C), 1328 (C=S), 1235 (C-O-C) cm^–1^; ^1^H-NMR (DMSO- *d* *_6_* , 400 MHz) ppm: *δ* = 12.25 (1H, bs, COO **H** ), 8.05 (1H, t, *J* = 0.8 Hz, phenyl H_2_), 7.87–7.85 (2H, m, phenyl H_5_+H_6_), 5.06 (2H, s, N-C **H** **_2_** -N), 3.34 (1H, bs, piperidine H_3_), 3.13 (1H, dd, *J* = 11.2 Hz, *J’* = 3.6 Hz piperidine H_2_), 2.91 (1H, dd, *J* = 11.2 Hz, *J’* = 3.6 Hz, piperidine H_2_^’^), 2.66 (2H, t, *J* = 10.0 Hz, piperidine H_6_), 1.78–1.63 (2H, m, piperidine H_4_), 1.47-1.31 (2H, m, piperidine H_5_); C^13^-NMR (100 MHz, DMSO) *δ* 177.4 ( **C** =O), 174.6 ( **C** =S), 156.6 ( **C** =N), 134.9 (phenyl **C** **_4_** ), 132.3 (phenyl **C** **_3_** ), 131.7 (phenyl **C** **_5_** ), 127.7 (phenyl **C** **_6_** ), 126.2 (phenyl **C** **_2_** ), 122.8 (phenyl **C** **_1_** ), 70.8 (N- **C** H_2_-N), 52.1 (piperidine **C** **_1_** ), 50.0 (piperidine **C** **_5_** ), 41.0 (piperidine **C** **_2_** ), 25.7 (piperidine **C** **_3_** ), 23.9 (piperidine **C** **_4_** ); Anal. Calcd. for C_15_H_15_Cl_2_N_3_O_3_S: C, 46.40; H, 3.89; N, 10.82; S, 8.26. Found: C, 46.15; H, 3.88; N, 10.86; S, 8.53.

##### 2.2.2.9. Ethyl 1-{[5-(3,4-dichlorophenyl)-2-thioxo-1,3,4-oxadiazol-3(2H)-yl]methyl}piperidine-4-carboxylate (5i)

White crystals, yield 85.13%, Mp 163.8°C; IR (KBr) ν_max_ : 2950 (Aromatic C-H), 1720 (C=O), 1611 (C=N), 1443–1421 (C=C), 1324 (C=S), 1241 (C-O-C) cm^–1^; ^1^H-NMR (DMSO- *d* *_6_* , 400 MHz) ppm: δ = 8.06 (1H, s, phenyl H_2_), 7.86 (2H, s, phenyl H_5_+H_6_), 5.04 (2H, s, N-C **H**_2_-N), 4.03 (2H, q, *J* = 6.8 Hz, COO-C **H**_2_-CH_3_), 3.02 (2H, d, *J* = 12 Hz, piperidine H_2_), 2.56 (2H, d, *J* = 10.8 Hz, piperidine H_6_), 2.24-2.21 (1H, m, piperidine H_4_), 1.81 (2H, d, *J* = 10.4 Hz, piperidine H_3_), 1.57–1.52 (2H, m, piperidine H_5_), 1.15 (3H, t, *J* = 6.8 Hz, COO-CH_2_-C **H**_3_); C^13^-NMR (100 MHz, DMSO) *δ* 177.5 ( **C** =O), 174.0 ( **C** =S), 156.6 ( **C** =N), 134.9 (phenyl **C** **_4_** ), 132.3 (phenyl **C** **_3_** ), 131.7 (phenyl **C** **_5_** ), 127.7 (phenyl **C** **_6_** ), 126.2 (phenyl **C** **_2_** ), 122.8 (phenyl **C** **_1_** ), 70.8 (N- **C** H_2_-N), 59.7 (O- **C** H_2_CH_3_), 49.1 (piperidine **C** **_1_** + **C** **_3_** + **C** **_5_** ), 28.7 (piperidine **C** **_2_** + **C** **_4_** ), 13.9 (O-CH_2_ **C** H_3_); Anal. Calcd. for C_17_H_19_Cl_2_N_3_O_3_S: C, 49.04; H, 4.60; N, 10.09; S, 7.70. Found: C, 48.64; H, 4.53; N, 10.00; S, 8.72.

##### 2.2.2.10. Ethyl 1-{[5-(3,4-dichlorophenyl)-2-thioxo-1,3,4-oxadiazol-3(2H)-yl]methyl}piperidine-3-carboxylate (5j)

White crystals, yield 75.51%, Mp 132.9°C; FT-IR (KBr) ν_max_ : 2934 (Aromatic C-H), 1727 (C=O), 1607 (C=N), 1414 (C=C), 1335 (C=S), 1180 (C-O-C) cm^–1^; ^1^H-NMR (DMSO- *d* *_6_* , 400 MHz) ppm: δ = 8.06 (1H, d, *J* = 1.6 Hz, phenyl H_2_), 7.87 (2H, t, *J* = 1.6 Hz, phenyl H_5_+H_6_), 5.05 (2H, s, N-C **H**_2_-N), 4.06 (2H, q, *J* = 7.2 Hz, COO-C **H**_2_CH_3_), 3.12 (1H, dd, *J* = 11.2 Hz, *J’* = 3.2 Hz, piperidine H_2_), 2.92-2.87 (1H, m, piperidine H_2_^’^), 2.72 (1H, t, *J* = 11.2 Hz, piperidine H_6_), 2.59–2.52 (2H, m, piperidine-H_6_’+H_4_), 1.76–1.63 (2H, m, piperidine H_3_), 1.47–1.35 (2H, m, piperidine H_5_), 1.17 (3H, t, *J* = 7.2 Hz, COO-CH_2_C **H**_3_); C^13^-NMR (100 MHz, DMSO) *δ* 177.5 ( **C** =O), 172.8 ( **C** =S), 156.7 ( **C** =N), 134.9 (phenyl **C** **_4_** ), 132.3 (phenyl **C** **_3_** ), 131.7 (phenyl **C** **_5_** ), 127.6 (phenyl **C** **_6_** ), 126.1 (phenyl **C** **_2_** ), 122.8 (phenyl **C** **_1_** ), 70.7 (N- **C** H_2_-N), 59.7 (O- **C** H_2_CH_3_), 51.9 ( **C** **_1_** , piperidine), 49.9 ( **C** **_5_** , piperidine), 40.9 ( **C** **_2_** **,** piperidine), 25.5 ( **C** **_3_** **,** piperidine), 23.7 ( **C** **_4_** **,** piperidine), 13.9 (O-CH_2_ **C** H_3_); Anal. Calcd. for C_17_H_19_Cl_2_N_3_O_3_S: C, 49.04; H, 4.60; N, 10.09; S, 7.70. Found: C, 48.64; H, 3.94; N, 9.99; S, 6.80.

##### 2.2.2.11. Ethyl 1-{[5-(3,4-dichlorophenyl)-2-thioxo-1,3,4-oxadiazol-3(2H)-yl]methyl}piperidine-2-carboxylate (5k)

White crystals, yield 32.05%, Mp 113.6°C; FT-IR (KBr) ν_max_ 2938 (Aromatic C-H), 1730 (C=O), 1448-1414 (C=C), 1321 (C=S), 1187 (C-O-C) cm^–1^; ^1^H-NMR (DMSO- *d* *_6_* , 400 MHz) ppm: δ = 8.04 (1H, d, *J* = 2.0 Hz, phenyl H_2_), 7.89 (1H, d, *J* = 8.0 Hz, phenyl H_6_), 7.84 (1H, dd, *J* = 8.8 Hz, *J’* = 2.0 Hz phenyl H_5_), 5.13 (2H, s, N-C **H**_2-_N), 4.09–4.02 (2H, m, COO-C **H**_2_-CH_3_), 3.69 (1H, t, *J* = 6.0 Hz, piperidine H_2_), 3.24–3.21 (2H, m, piperidine H_6_), 1.77–1.67 (2H, m, piperidine H_3_), 1.51-1.48 (2H, m, piperidine H_5_), 1.40–1.30 (2H, m, piperidine H_4_), 1.18 (3H, t, *J* = 6.8 Hz, COO-CH_2_-C **H** **_3_** ); C^13^-NMR (100 MHz, DMSO) *δ* 177.1 ( **C** =O), 172.5 ( **C** =S), 156.3 ( **C** =N), 134.9 (phenyl **C** **_4_** ), 132.3 (phenyl **C** **_3_** ), 131.8 (phenyl **C** **_5_** ), 127.5 (phenyl **C** **_6_** ), 126.0 (phenyl **C** **_2_** ), 122.7 (phenyl **C** **_1_** ), 68.9 (N- **C** H_2_-N), 59.9 (O- **C** H_2_CH_3_), 48.2 (piperidine **C** **_1_** + **C** **_5_** ), 29.0 (piperidine **C** **_2_** ), 24.8 (piperidine **C** **_4_** ), 20.9 (piperidine **C** **_3_** ), 13.9 (O-CH_2_ **C** H_3_); Anal. Calcd. for C_17_H_19_Cl_2_N_3_O_3_S: C, 49.04; H, 4.60; N, 10.09; S, 7.70. Found: C, 49.13; H, 4.72; N, 10.20; S, 7.74.”

### 2.3. Biological assays

#### 2.3.1. Antimicrobial activity

##### 2.3.1.1. Disc diffusion method

Dimethylsulfoxide (DMSO) was used to dissolve and prepare the synthesized compounds with a concentration of 10 mg mL^–1^. The lyophilized compounds sterilized by filtration via 0.45 mm millipore filters. Disc diffusion method was performed by using 100 mL of suspension containing 108 colony forming units (CFU) mL^–1^ of bacteria, 106 CFU mL^–1^ of yeast and 104 spore mL^–1^ of fungi spread on nutrient agar (NA), sabour dextrose agar (SDA), and potato dextrose agar (PDA) medium, in sequence. A total of 15 mL of each synthesized compounds (300 mg/disc) at the concentration of 10 mg mL^–1^ were impregnated to the discs (6 mm in diameter). DMSO impregnated discs were used for negative controls. The compounds and negative controls were located in the inoculated agar. In order to determine the sensitivity of one strain/isolate standard, ofloxacin and nystatin were used as positive references for bacterial and fungus-yeast strains, respectively. The incubation at 37°C of inoculated plates took 24 h for bacterial strains, 48 h for yeast and 72 h for fungi isolates. The incubation of plant related microorganisms were held at 27°C, differently [31].

#### 2.3.2. Cytotoxic activity

The human cancer cell lines were grown in Dulbecco’s Modified Eagle’s Medium (DMEM), with 10% fetal bovine serum (FBS) and 1% penicillin. They were incubated in 37 °C incubators containing 5% CO_2_ and 95% air. Cancer cells (range of 2000 cells/well to 5000 cells/well) were inoculated into 96-well plates in 200 μL of media and incubated in 37 °C incubators containing 5% CO_2_ and 95% air. After a 24 h incubation period, one plate for each cell line was fixed with 100 μL of 10% ice-cold trichloroacetic acid (TCA). This plate represents the behavior of the cells just prior to compound treatment and is accepted as the time-zero plate. The compounds to be tested were solubilized in dimethyl sulfoxide (DMSO) to a final concentration of 40 mM and stored at +4°C. While treating the cells with the compounds, the corresponding volume of the compound was applied to the cell to achieve the desired drug concentration and diluted through serial dilution (40, 20, 10, 5, 2.5 µM). After drug treatment, the cells were incubated in 37 °C incubators containing 5% CO_2_ and 95% air for 72 h. Following the termination of the incubation period after drug treatment, the cells were fixed with 100 μL 10% ice-cold TCA and incubated in the dark at +4°C for 1 h. Then, the TCA was washed away with ddH_2_O five times and the plates were left to air dry. In the final step, the plates were stained with 100 μL of 0.4% SRB (cat.86183-5 g from Sigma) solution in 1% acetic acid solution. Following staining, the plates were incubated in dark for 10 min at room temperature. The unbound dye was washed away using 1% acetic acid and the plates were left to air dry. To measure the absorbance results, the bound stain was then solubilized using 200 μL of 10 mM Tris-Base. Camptothecin was the positive control and 5-Fluorouracil (5-FU) was standard drug for the cytotoxic effect. The OD values were obtained at 515nm [32].

### 2.4. In silico chemo-informatic and toxicity measurements

For determination of drug-like physicochemical, pharmacokinetics, and toxicity parameters, a combination of various online screening tools were used, which included MedChem Designer 5.5 (MedChem Designer, version 5.5.0.112019), Chem&BioDraw 12.0 (ChemDraw version 12.0.2.10762019), SwissAdme (Swiss Institute of Bioinformatics2013_http://www.swissadme.ch/). Toxicity prediction of these newly synthesized compound **5a-5k** series were retrieved from Lazar software (Version 1.4.2) which is a web-based application as https://lazar.in-silico.ch [33].

## 3. Results and discussion

### 3.1. Chemistry

5-(3,4-Dichlorophenyl)-3-[(substitutedpiperidine)methyl]-1,3,4-oxadiazole-2( *3H* )-thione derivatives ( **5a-5k** ) were prepared via Mannich reaction. According to chemical procedure of piperidine, derivatives were reacted with (3,4-dichlorophenyl)-1,3,4-oxadiazole-2( *3H* )-thione ( **4** ) group in alcoholic media (Figure). Compounds **4** , **5a-5k** were characterized by FT-IR, ^1^H NMR, ^13^C NMR spectroscopy, and purity of compounds were checked with elemental analysis. All results of spectral and elemental analysis were found compatible with literature data [34–37]. Compounds **4, 5a-5k** were tested for their antimicrobial and cytotoxic properties. 

**Figure:**
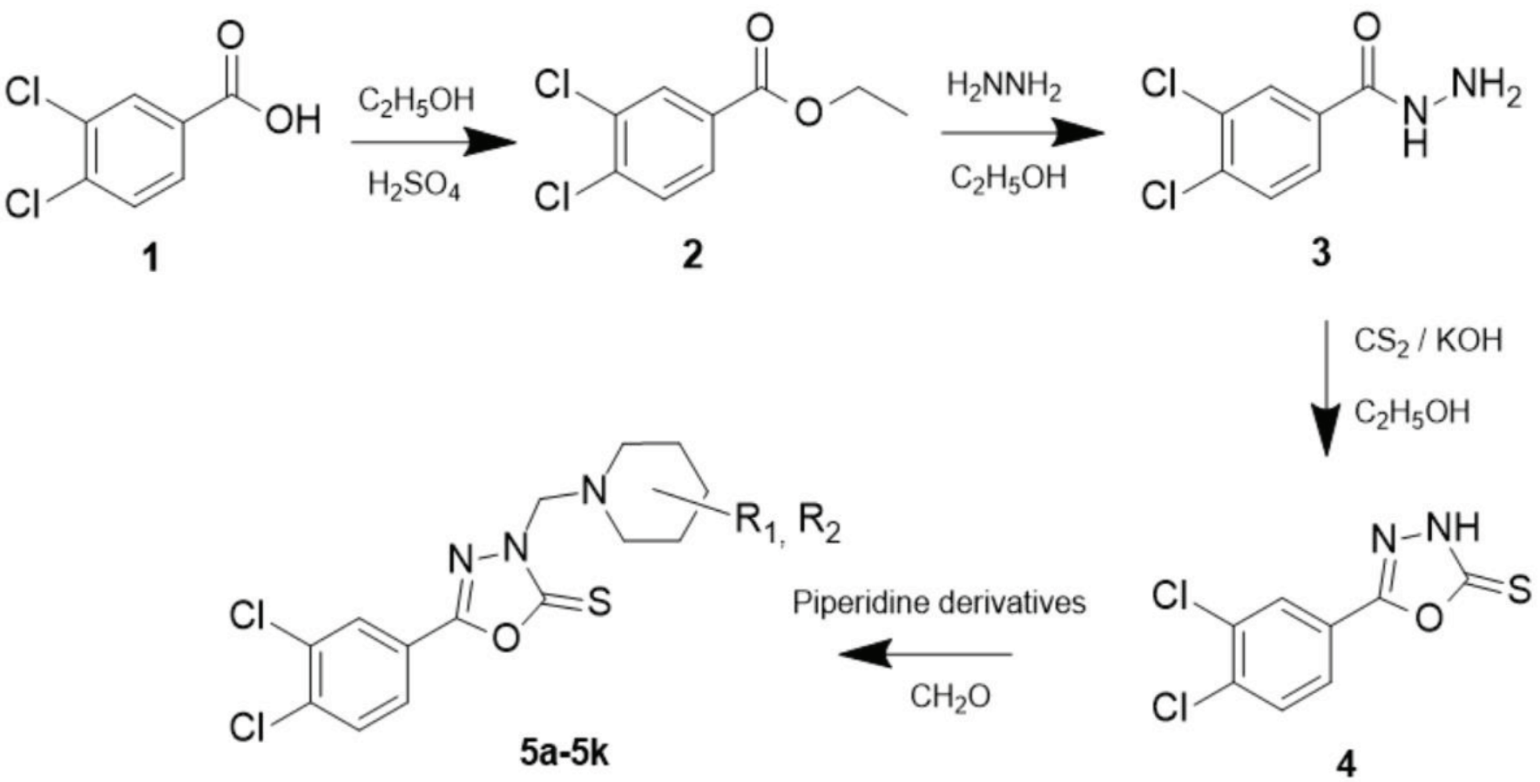
Synthesis of (3-4-dichlorophenyl)-1,3,4-oxadiazole-2(3H)-thione derivatives.

The FT-IR spectrum of compounds displayed a strong band in range of 3080–2900 cm^-1 ^which assigned to aromatic carbon-hydrogen sp^2^ hybridizations in common for all compounds. Imine (C=N) and thione (C=S) groups in 1,3,4-oxadiazole-2( *3H* )-thione structure, generated two characteristic signals approximately at 1610 and 1330 cm^–1^. Compounds **5c, 5g-5k** had an extra sharp signal around 1740–1680 cm^–1^, which corresponded to the carbonyl group, and compound 5d showed a spesific band at 2238 cm^–1^, which was claimed as nitrile group. 

^1^H NMR spectra of compounds **4, 5a-5k** demonstrated hydrogen signals of aromatic structures in the range of 8.00–7.00 ppm. Two proton integrationed and singlet coupled signal in the range of 5.13–5.01 ppm values was a strong evidence for methylene bridge protons between 1,3,4-oxadiazole and piperidine moieties which obtained via *Mannich* reaction procedure. Variable but compatible integrated signals between 3.60–1.30 ppm confirmed different piperidine protons for each compound. Compound **5c** have an acetyl group and preserved a singlet signal in 1.84 ppm for alpha protons. Signals for compounds **5i-5k** , which have different positioned ethyl ester groups on piperidine moiety emerged at 4.06–4.02 for methylene protons (-COOC **H**_2_-) and 1.18–1.15 ppm values for methyl protons (-COOCH_2_C **H**_3_). Integration and multiplicity of signals for all compounds were compatible with literature data. 

^13^C NMR spectra of compounds **4, 5a-5k** preserved two characteristic signals related to thione ( **C** =S) and imine ( **C** =N) groups at 179 and 158 ppm values originated from 1,3,4-oxadiazole-2( *3H* )-thione structure. Carbon signal of ketone carbonyl in compound **5b** , carboxy and ester carbonyl in compounds **5g-5k** were appeared at different ppm values due to different chemical environments of carbonyl functional groups. While ketone carbonyl carbon of **5c** showed signal at 208 ppm, carboxy and ester carbonyl carbons of compound **5g-5k** indicated their carbonyl carbon signals in the range of 179–174 ppm. Due to shielded-deshielded properties of carbon atoms in magnetic field of ^13^C NMR, signal of ketone carbonyl in **5c** occured in downfield region while carboxylic acid ( **5g, 5h** ) and ester ( **5i-5k** ) carbonyl carbons shifted through upfield part of the scale. In this direction, moderately shielded aromatic structures in compounds **4, 5a-k** gave their spesific signals in the range of 138–122 ppm. ^13^C NMR signals of remaining carbons associated with methylene and piperidine groups were observed at 70–65 and 69–32 ppm. 

### 3.2 Biological evaluation 

#### 3.2.1. Antimicrobial activity

Antimicrobial activity was tested by measuring the zone of inhibition against test organisms with disc diffusion assay method, and results were summarized in Table 1 and Table 2 with positive control ofloxacin. Eleven compounds were screened for their antibacterial activity against three gram-negative ( *E. coli, P. aeruginosa, P. vulgaris* ) and sixteen gram-positive bacterial strains. ( *Staphylococcus spp, Micrococcus spp, Bacillus spp* ). They were also evaluated for their antifungal potential against six fungal strains ( *Aspergillus spp, F. oxysporium, B. cinerea, Penicillium, Candida spp* ) and antiyeast activity against three yeast strains ( *K. marxianus, P. membranaefaciens, S. occidentalis* ). Ofloxacin and nystatin were used as positive controls. Antimicrobial data of compounds and reference drugs were given in Tables 1 and Table 2.

**Table 1:** Antibacterial activities of newly synthesized compounds.

Test microorganisms	4	5a	5b	5c	5d	5e	5f	5g	5h	5i	5j	5k	Ofloxacin	
Escherichia coli	-	-	-	-	-	-	-	-	-	-	-	-	22	Zone of inhibition in mm
Pseudomonas aeruginosa	-	-	-	-	-	-	-	-	8	-	-	-	22	
Pseudomonas vulgaris	14	14	12	12	10	14	10	10	12	12	14	14	23	
Staphylococcus aureus	10	10	12	10	12	14	14	12	12	12	14	12	22	
Staphylococcus cohnii	12	10	20	14	14	11	14	14	14	12	14	12	24	
Micrococcus lylae	14	12	10	10	10	10	10	11	12	11	11	9	13	
Micrococcus luteus	14	9	12	9	9	10	9	12	12	9	10	9	13	
Bacillus megaterium	14	12	15	14	11	12	16	14	15	16	12	12	26	
Bacillus lentimorbus	24	24	22	20	18	16	14	18	20	16	18	16	26	
Bacillus subtilis	15	15	12	12	12	17	15	14	17	16	15	15	21	
Bacillus licheniformis	22	13	20	15	14	13	14	17	14	13	14	13	25	
Bacillus pumilus	20	10	15	14	14	15	18	20	19	13	16	12	20	
Bacillus mycoides	20	12	23	20	15	17	20	20	22	15	19	19	25	
Bacillus cereus	14	12	16	16	14	14	15	15	16	14	14	13	15	
Bacillus ehimensis	30	20	32	24	18	20	30	26	28	24	28	22	21	
Bacillus thuringiensis	14	13	18	15	14	12	13	15	16	12	12	11	14	
Bacillus sphaericus	9	-	11	8	8	10	10	-	10	8	10	10	17	
Bacillus marinus	24	15	22	12	14	20	20	22	22	20	24	20	30	
Bacillus laevolacticus	26	12	21	14	15	17	20	17	20	13	19	17	30	

**Table 2:** Antifungal and antiyeast activities of newly synthesized compounds.

Test microorganisms	4	5a	5b	5c	5d	5e	5f	5g	5h	5i	5j	5k	Nystatin	
Aspergillus spp	9	-	-	-	-	-	-	-	-	-	-	-	12	Zone of inhibition in mm
Fusarium oxysporium	12	8	10	9	8	9	9	10	13	8	12	9	14	
Botrytis cinerea	17	12	15	10	9	10	10	11	12	10	10	12	25	
Penicillium spp	19	-	12	-	-	1	-	14	16	-	13	11	14	
Candida albicans	14	10	16	14	12	14	16	14	16	14	12	12	20	
Candida parapsilosis	14	8	14	14	12	12	11	12	14	11	13	12	20	
Kluyveromyces marxianus	12	11	14	12	11	11	12	14	16	14	15	15	20	
Pichia membranaefaciens	15	12	18	12	12	16	16	18	18	13	15	15	18	
Schwanniomyces occidentalis	17	10	20	14	10	14	16	13	20	12	14	14	20	

In vitro disc diffusion test was carried out to evaluate newly synthesized compounds ( **4, 5a-k** ) for their antibacterial activities towards pathogenic gram-positive and gram-negative bacteria, and ofloxacin was used as positive control under the same conditions. As shown in Table 1, compound series displayed serious inhibition of growth (mm) in certain bacterial strains. Especially compounds **5b, 5c, 5f-5k** showed considerable antibacterial activity against gram-positive *Bacillus spp* . when compared to ofloxacin, while inhibition of growth values (mm) of other compounds in the series were either equal or lower than reference molecule. In vitro antibacterial screening results also revealed that, for all bacterial strains, lower inhibition values than reference material meaned as an indicator of antibacterial inability especially for compounds **5a, 5d** and **5e** (Table 1). None of compounds didn’t show any antibacterial activity against *E.coli* and *P. Aeruginosa* but compounds **5b, 5f, 5g, 5h** and **5j** showed statistically significant antibacterial activity against *B. ehimensis* when they compared with ofloxacin.

Chemical nature of substitution pattern related to piperidine derivatives of 5-(3,4-dichlorophenyl)-1,3,4-oxadiazole-2( *3H* )-thione compounds was important point to establish biological activity-functional group relationship. Therefore, newly synthesized molecules were designed to emphasize this correlation. As a comparable result, compounds **5a, 5b, 5c** and **5e** , which have a common phenyl group, showed different biological responses for *B. ehimensis* strains. Other substituents and phenyl groups that were located on the fourth position piperidine moiety had significant functionality differences for activity. Based on above part, strong electron-donating hydroxyl containing compound **5b** , weak electron-withdrawing acetyl containing **5c** , strong electron-withdrawing nitrile containing **5d** , and only phenyl substituted piperidine containing compound **5a** were good examples to elucidate this phenomenon. According to structure-activity relationship (SAR) studies in literature **,** electron-donating groups provide an elevation of inhibitor level against spesific bacterial strains [38]. The results obtained in parallel with this information was compound **5b** , which had high level of antimicrobial property specifically against *B. ehimensis* due to its electron-donating hydroxyl moiety. In addition of these four compounds ( **5a-d** ), morpholine containing compound 5f was also found more active than reference drug on *B. ehimensis* strain. Heteroatoms in morpholine and their bonding capasities with active site of bacteria were serious indicators for biologic activity of 5f **,** which was an open point for further studies on heterocyclic structure substituted piperidine rings. Besides, substituent variation and their effects on biologic acitiy, structural design was modulated also to generate position effect on activity. So, on piperidine moiety, differently located carboxylic acid containing compounds **5g, 5h** and differently positioned ethyl ester containing compounds **5i** , **5j** and **5k** were added to series. Results clearly claimed that position differences of one substituent on piperidine did not cause a significant difference for their antimicrobial response (Table 1). 

In vitro antimicrobial activity of compounds **4, 5a-k** were further assessed in terms of antifungal and antiyeast activities relative to nystatin according to disc diffusion assay method **.** The results were presented in Table 2 and a review of data revealed that all compounds were possessed moderate activities against *Candida spp* and no inhibition against *Aspergillus spp* except compound **4** . The best and comparable results were obtained against *Penicillium sp.* and *F. oxysporium* for compounds **4, 5g,** and **5h** . According to antiyeast activity profile, compounds showed weak to moderate activity against *K. Marxianus* wherease compounds **5b, 5g** and **5h** were equipotent against *P. membranaefaciens* and *S. occidentalis* when they were compared with nystatin. Also compound **5b** , which has strong electron-donating hydroxyl group showed the best activity against all fungal and yeast strains according to other phenyl containing compounds **5a, 5c,** and **5d** . This unclear activity profile and lipophilicity relationship might be seen as described in previous study [39] **.** Due to the consistent results generated from antibacterial, antifungal and antiyeast activities, synthesized compounds were further analysed for their cytotoxic activities on certain cancer cell lines.

#### 3.2.2. Cytotoxicity study 

All synthesized target compounds **5a-5k** were screened for their cytotoxic activity against three cancer cell lines: colon (HCT116), breast (MCF7), and liver (HUH7) with sulphorhodamine B (SRB) assay in triplicate aplication where 5-flourouracil (5-FU) was used as positive control. The IC_50_ values obtained for these compounds were shown in Table 3. 

**Table 3:** IC_50_ value for tested compounds 5a-5k against cancer cell lines.

	5a	5b	5c	5d	5e	5f	5g	5h	5i	5j	5k	5-FU	
HCT116*	NI	NI	NI	18.2	33.3	47.9	44.1	80.4	NI	NI	13.9	30.7	IC50 (µM)
MCF7*	NI	40.9	NI	NI	26.8	NI	29.0	37.4	NI	45.0	25.2	3.5	
HUH7*	NI	27.5	11.8	10.1	18.3	16.4	14.0	34.6	17.8	15.2	11.9	18.78	

*All the experiments were conducted in triplicate (1 < R^2^ < 0.8). NI: no inhibition.

The results of cytotoxicity studies revealed that activities of the compounds were not impressive against colon and breast cancer cells, all of the compounds showed cell viability with IC_50_ values ranging from 13.9–80.4 µM concentrations. It was noteworthy that the cytotoxic effects were more pronounced against liver carcinoma cell line, HUH7. Most of the compounds of the series ( **5c-5g** and **5i-5j** ) have better IC_50_ values than 5-FLU (IC_50 _= 18.78 µM) and also compound **5d** possessed 10.1 µM value, which represents good druggable cytotoxic activity. 

Our results indicate that the addition of phenyl and carbonyl group as substituents enhances the antimicrobial activity of the prepared compounds. It indicates that the structural differences is an important factor for the activity. It is notable, also, that phenyl and carbonyl substitutions in compounds **5c, 5d, 5f, 5g, 5i-5k** lead to not only to an increase in the antimicrobial activity on certain bacterial and fungal strains, but also to a significant level of anticancer activity against liver carcinoma cell line (HUH7). According to the preliminary antimicrobial activity, compound **5b** showed the best inhibitor activity against *Bacillus spp* strains, and compound **5d** was evaluated to have strongest cytotoxic activity against liver carcinoma cells (HUH7). A total analysis of the antibacterial, antifungal, and antiyeast activity revealed that newly synthesized compounds were really active against *Bacillus cereus* , *Bacillus ehimensis* and *Bacillus thuringiensis* species *.*

### 3.3. In silico chemo-informatic and toxicity measurements

The chemo-informatic features of newly synthesized molecules were evaluated by computational tools. According to in silico data, compounds **5a-5k** showed acceptable consequences for Lipinski’s rule of five (RO5) analysis; molecular weight (MW) (<500 dalton), hydrogen bond acceptor (HBA) (<10), hydrogen bond donor (HBD) (<5) and logP (<5) values [40]. In the situation of one deviation, corresponds to poor absorption of compounds. However, there are plenty of examples are available for RO5 violation amongst the existing drugs [41,42]. Furthermore, the polar surface area (PSA) of a molecule is defined as the surface sum over all polar atoms, primarily oxygen and nitrogen with their attached hydrogen atoms. The PSA value of a molecule reflects the ability to permeate cells, which is used for drug’s optimization. Previous researches showed the standard value of PSA as <89 A^2^ [43] in which this measure is supported by our newly synthesized compound **5a-5k** serie. On the other hand, number of rotatable bond is a measurement for molecular flexibility and is significant in determining oral bioavailability of the compounds (rule of three-number of rotatable bonds ≤ 3), which explains oral usage of compound **5a-5k** might be decrease bioavailibility (Table 4) [44].

**Table 4:** Chemo-informatic data of compound 5a-5k.

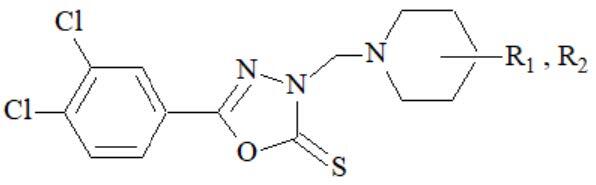								
Compound	R1	R2	MW (Da)	HBA	HBD	RB	LogP	PSA
5a	4-phenyl	-	420.36	4	0	4	6.30	28.07
5b	4-phenyl	4-hydroxy	436.35	5	1	4	5.10	48.30
5c	4-phenyl	4-acetyl	462.39	5	0	5	5.69	45.14
5d	4-phenyl	4-cyano	445.36	5	0	4	6.32	51.86
5e	4-benzyl	-	434.38	4	0	5	6.72	28.07
5f	4-morpholine	-	429.39	6	0	4	3.60	40.54
5g	4-carboxylic acid	-	388.27	6	1	4	3.95	65.37
5h	3-carboxylic acid	-	388.27	6	1	4	4.09	65.37
5i	4-(ethyloxycarboxyl)	-	416.32	6	0	6	4.55	54.37
5j	3-(ethyloxycarboxyl)	-	416.32	6	0	6	4.69	54.37
5k	2-(ethyloxycarboxyl)	-	416.32	6	0	6	4.81	54.37

*HBA (Hydrogen Bind Acceptor) and HBD (hydrogen bind donor) values of compound 5a-5k were calculated by MedChem Designer 5.5. *MW (molecular weight), logP and PSA (polar surface area) values of compound 5a-5k were calculated by Chem & Bio Draw 12.0. *RB (rotatable bond) value of compound 5a-5k were calculated by SwissAdme.

Toxicity prediction is a tool is an useful method in the drug discovery due to many of the newly synthesized potential candidates had failed in clinical trial evaluation because of some pharmacokinetics and toxicity problems. In silico predictions are improved to overcome such scenario in this detailed process. Meanwhile, toxicity predictions were clearly revelead that all our novel compounds are seemed to have no predictable central nervous system side effects, carcinogenicity, and mutagenicity (Table 5).

**Table 5:** In silico predicted toxicity measurements of compound 5a-5k.

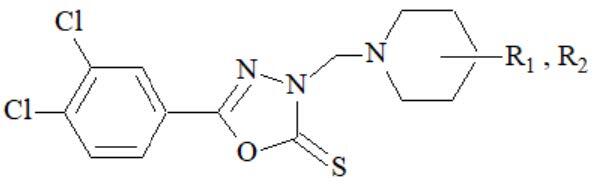					
Compound	R1	R2	BBBP	CG	MG
5a	4-phenyl	-	Non-penetrating	Non-carcinogenic	Non-mutagenic
5b	4-phenyl	4-hydroxy	Penetrating	Non-carcinogenic	Non-mutagenic
5c	4-phenyl	4-acetyl	Non-penetrating	Non-carcinogenic	Non-mutagenic
5d	4-phenyl	4-cyano	Non-penetrating	Non-carcinogenic	Non-mutagenic
5e	4-benzyl	-	Non-penetrating	Non-carcinogenic	Non-mutagenic
5f	4-morpholine	-	Non-penetrating	Non-carcinogenic	Non-mutagenic
5g	4-carboxylic acid	-	Non-penetrating	Non-carcinogenic	Non-mutagenic
5h	3-carboxylic acid	-	Non-penetrating	Non-carcinogenic	Non-mutagenic
5i	4-(ethyloxycarboxyl)	-	Non-penetrating	Non-carcinogenic	Non-mutagenic
5j	3-(ethyloxycarboxyl)	-	Non-penetrating	Non-carcinogenic	Non-mutagenic
5k	2-(ethyloxycarboxyl)	-	Non-penetrating	Non-carcinogenic	Non-mutagenic

* BBBP: Blood brain barrier penetration; CG: Carcinogenicity; MG: Mutagenicity.

## 4. Conclusion 

In summary, we report the efficient synthesis, characterization, antimicrobial and cytotoxic activity evaluation of new compound series which contain different substituted piperidine bearing 1,3,4-oxadiazole-2( *3H* )-thione structures. According to biological consequences, phenyl and carbonyl group that substituted to piperidine ring seemed to have supportive property on antimicrobial activity of the novel compounds. Some compounds like 5 **c, 5d, 5f, 5g, 5i-5k** that contain phenyl and carbonyl group revealed not only antimicrobial effect on certain bacterial and fungal strains but also significant level of anticancer activity against liver carcinoma cell line (HUH7). Especially compound **5b** showed the best inhibitor activity against *Bacillus spp* whereas compound **5d** represent valuable effect against liver carcinoma cells (HUH7). Besides, evaluating biological properties, compounds were predicted for the chemo-informatic and possible toxicity features within some software programmes. Synthesized molecules were calculated as to qualified the criteria to be a drug as per Lipinski Rule of Five and in silico predicted toxicology results were seemed as all of them have no mutagenic or carcinogenic profiles. 

## Supplementary Information

^1^H and ^13^C NMR spectra of all compounds, 5a-5k, are given as supplementary information at www.ias.ac.in/chemsci. 
